# Sense of coherence as a protective factor in sleep and awake bruxism effects on adolescents quality of life

**DOI:** 10.1590/1807-3107bor-2026.vol40.022

**Published:** 2026-05-18

**Authors:** Jessica Klöckner Knorst, Eduardo Machado, Eduarda da Silveira Borstmann, Thiago Machado Ardenghi

**Affiliations:** (a) Universidade Federal de Santa Maria – UFSM, Pos-Graduation Program in Dentistry, Santa Maria, RS, Brazil.; (b) Universidade Fedetal de São Paulo – Unifesp, Department of Psychobiology, São Paulo, SP, Brazil.

**Keywords:** Adolescent, Bruxism, Oral Health, Quality of Life, Sense of Coherence

## Abstract

This study aimed to assess whether the sense of coherence (SOC) moderates the association between self-reported sleep bruxism (SB) and self-reported awake bruxism (AB) on oral health-related quality of life (OHRQoL). A cross-sectional analysis was conducted with a random sample of 406 adolescents aged 11 to 17 years from Southern Brazil. OHRQoL was measured using the Child Perception Questionnaire (CPQ11-14). Both SB and AB were assessed by self-report (weekly frequency). SOC was measured using the 13-item Sense of Coherence Scale (SOC-13). Demographic, socioeconomic, and clinical variables were included as potential confounders. Poisson regression models were used to test interaction effects. Adolescents with bruxism behaviour—either SB or AB—presented significantly higher CPQ11-14 scores, indicating poorer OHRQoL. SOC moderated this association: adolescents with SB and low SOC reported worse OHRQoL than those with high SOC. Similar results were observed for AB. In addition, the moderating effect was stronger for AB, with a greater difference in predicted CPQ11-14 scores across SOC levels. These findings suggest that SOC may buffer the psychosocial and functional effects of bruxism. Strengthening SOC could be a promising avenue for interventions aimed at minimizing the quality-of-life burden of bruxism in adolescents.

## Introduction

In recent decades, there has been an evident rise in the number of individuals diagnosed with harmful behaviors that affect the stomatognathic system.^1–2^ This trend is particularly apparent among children and adolescents, with an increasing identification of bruxism signs and symptoms. These findings have raised concerns among health professionals, as bruxism presents diagnostic and therapeutic challenges and may negatively affect esthetics and oral function.^3^

Bruxism is defined as masticatory muscle activity that occurs during sleep (rhythmic or non-rhythmic) or wakefulness (characterized by tooth contact, bracing, or thrusting).^4^ Bruxism is a motor behavior and it is not considered a movement or sleep disorder. The current consensus recommends specifying the assessment method: self-report (subject-based), clinical examination (clinically-based), or instrumental measures (device-based).^4^

The prevalence of bruxism among adolescents ranges from 3% to 49%,^5,6^ highlighting its relevance in this age group. Bruxism can negatively impact individuals’ daily lives, causing pain, discomfort during mastication, and esthetic concerns.^7–9^ Given its multifactorial consequences, bruxism during adolescence may impair well-being and oral health-related quality of life (OHRQoL). The concept of OHRQoL refers to individuals’ comfort and satisfaction with their oral health in performing daily activities such as eating, sleeping, and social interaction.^10–11^ Previous studies have demonstrated that bruxism can affect various domains of OHRQoL.^7–9,12–14^

Studies have tried to identify individual and social attributes that may serve as a resource for resilience and improve the perceived oral health of individuals, such as the sense of coherence (SOC).^15–17^ SOC is a central concept in Antonovsky's salutogenic theory, defined as a global orientation that reflects an individual's ability to perceive life as comprehensible, manageable, and meaningful.^18^ Individuals with a high level of SOC tend to cope more effectively with stressors and challenges, promoting better health and well-being.^15,18^ Given its influence on how people handle adversity, SOC may act as a moderating factor in the relationship between sleep bruxism and OHRQoL. Since sleep bruxism has been associated with negative oral health outcomes,^8–10^ individuals with higher SOC may better interpret and manage these impacts, mitigating their negative consequences.

Therefore, it is plausible to hypothesize that adolescents with higher levels of SOC may experience a weaker association between bruxism and OHRQoL, while those with lower SOC may be more vulnerable to its negative impacts. To the best of our knowledge, no previous study has investigated the moderating role of SOC in the relationship between both sleep and awake bruxism and OHRQoL. This study aimed to fill that gap. A better understanding of SOC's influence may support its inclusion as a valuable resource in the multifaceted management of bruxism.

## Methods

### Reporting and ethical aspects

This study is reported in accordance with the STROBE (Strengthening the Reporting of Observational Studies in Epidemiology) guidelines. Ethical approval was obtained from the Research Ethics Committee of the Federal University of Santa Maria (CAAE 54257216.1.0000.5346), and authorization was granted by both municipal and regional education authorities. Written informed consent was obtained from all parents or legal guardians, and adolescents provided written assent prior to participation.

### Sample

This cross-sectional study was nested within a 13-year cohort conducted in Santa Maria, a city in southern Brazil with a population of 271,633 inhabitants according to the 2022 census by the Brazilian Institute of Geography and Statistics (IBGE).^19^ Baseline data collection began in 2010 (T1) during the National Children's Multivaccination Day, using a systematic sample of 639 preschool children who attended health centers equipped with dental chairs (n = 15), distributed across various neighborhoods and administrative regions of the city. Participants were reassessed on four occasions, with the present study utilizing data from the most recent follow-up conducted in 2022–2023, totaling 13 years of follow-up (Figure). Further methodological details of the cohort have been published previously.^20^

**Figure:**
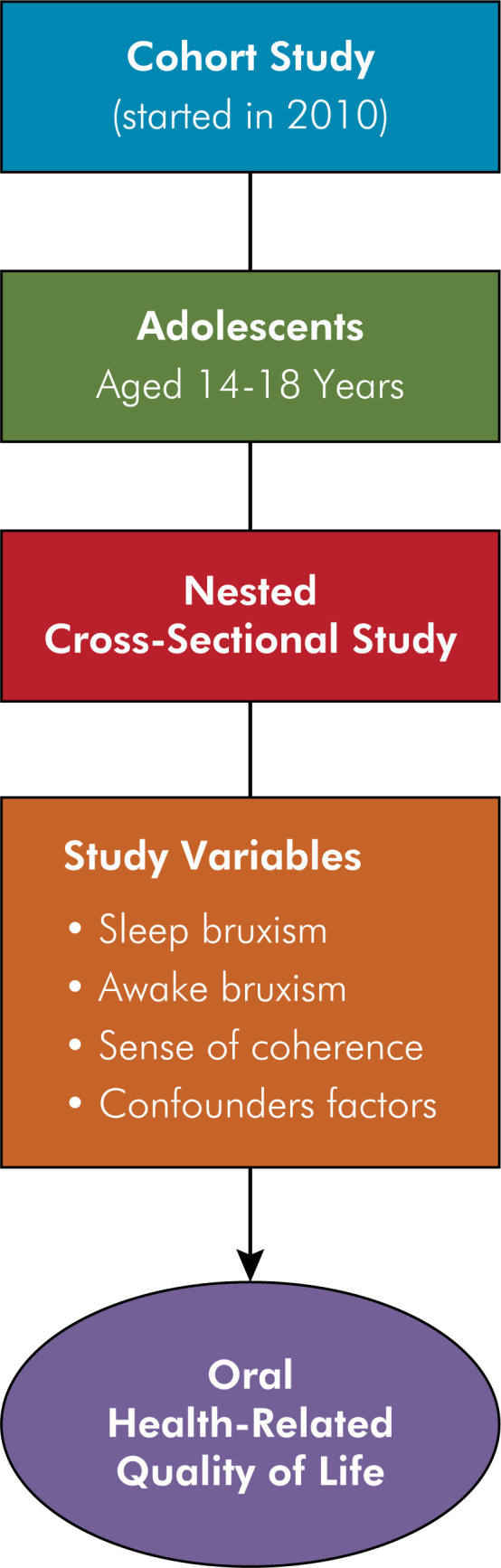
Schematic diagram of the study design.

A post-hoc power analysis was conducted using G*Power software to determine the statistical power of the study. The analysis was based on the following parameters: a significance level of 0.05, an effect size of 0.6 (considered a medium effect)^21^, a sample size of 406 adolescents, and a regression model with seven predictors (Model 1= SB; Model 2 = AB). The results indicated that the study had a power of 100% to detect differences for both models.

### Data collection and variables

Data collection was conducted by a team of eight previously trained dentists. Information on various variables was obtained through structured questionnaires and face-to-face interviews. Clinical examinations were performed in appropriate settings using a dental mirror, probe, and natural light. The examiners were previously trained and calibrated, with inter and intra-examiner Kappa coefficients ranging from 0.70 to 0.96. Participants were examined individually using gauze, a CPI probe ("ball point"), and a dental mirror. Both examinations and questionnaires were administered either at the adolescents’ schools or in their homes, depending on their availability.

The outcome of interest (OHRQoL) was assessed using the short form of the Child Perceptions Questionnaire (CPQ11-14),^22^ which was completed by the adolescents. The instrument comprises 16 items divided into four domains: oral symptoms, functional limitations, emotional well-being, and social well-being. Each item is rated on a 5-point Likert scale: (0) never, (1) once or twice, (2) sometimes, (3) often, and (4) every day or almost every day. The total score ranges from 0 to 64, with higher scores indicating a greater negative impact of oral health on quality of life.

Subject-based SB and AB were assessed following existing literature and consensus guidelines that emphasize frequency as a key assessment criterion.^4,13,23^ SB was evaluated by asking the adolescent the question: "Do you grind your teeth while sleeping?"; AB was evaluated by asking the question: "Do you clench or grind your teeth while awake?". The patients were instructed to answer ‘yes’ if they considered their habit to be frequent enough to be clinically relevant (e.g., frequency of more than three times a week and/or several hours per day), as suggested by previous literature,^23^ for each variable.

Participants completed the 13-item short version of the Sense of Coherence Scale (SOC-13), originally developed by Antonovsky (1987) and later translated, adapted, and validated for use in Brazil.^18,24^ The SOC-13 assesses an individual's sense of coherence across three components: comprehensibility, manageability, and meaningfulness. Each item is scored on a 5-point Likert scale, with response options varying by item and coded from 1 to 5. Total scores range from 13 to 65, with higher scores indicating a stronger SOC. For analytical purposes, SOC-13 scores were dichotomized at the median into low (≤ 38) and high (> 38) SOC.^24^

Demographic and socioeconomic variables were collected using a structured questionnaire and included as covariates in the analysis. Demographic data comprised sex (male or female), age (in years), and self-reported skin color, categorized as White or Black/Brown/Yellow.^19^ This categorization was adopted because of the relatively small number of participants in each non-White group, which limited the use of more detailed categories in the regression analyses. Household income was reported in Brazilian reals and categorized based on the Brazilian minimum wage (BMW) as either ≤1 BMW or >1 BMW, with one BMW approximately equivalent to 200 US dollars. Dental caries was assessed according to the International Caries Detection and Assessment System (ICDAS) criteria.^25^ For analytical purposes, caries experience was dichotomized as presence (ICDAS codes 3, 5, or 6) or absence (ICDAS codes 0, 1, 2, or 4) of cavitated lesions.

### Data analysis

Data analysis was performed using STATA 17.0 (StataCorp, College Station, TX, USA). Descriptive statistics were used to characterize the sample. The outcome was overall CPQ11-14 score. The moderating effect of SOC on the association between SB, AB, and CPQ11–14 was assessed by Poisson regression analyses considering multiplicative scales. The interaction was assessed by two distinct models: 1) presence or absence of SB according to each SOC level; 2) presence or absence of AB according to each SOC level. Demographic, socioeconomic, and clinical variables with a p ≤ 0.20 in unadjusted analyses were included as potential confounders in the adjusted models. Results are expressed as rate ratio (RR) and 95% confidence intervals (95% CI).

Subsequently, when the hypothesized moderation effects reached statistical significance, a simple slope test was conducted to estimate the predicted values at each level of the moderator. This approach allowed for the calculation of the conditional effect of X (Model 1=SB; Model 2=AB) on Y (CPQ11–14) across different SOC levels^26,27^. A contrast test was performed to assess differences in predicted values and CI among exposed groups. A significance level of 0.05 was adopted.

## RESULTS

A total of 406 adolescents were included in the study. The sample was balanced by sex, with a slight predominance of girls (51.5%), and the majority of participants were 16 or 17 years old. Most adolescents self-identified as White (77.1%) and reported a household income greater than one Brazilian minimum wage (77.7%). More than half of the sample (57.7%) exhibited low SOC. Regarding oral health characteristics, 10.9% of adolescents reported SB and 31.1% reported AB. The prevalence of untreated dental caries was 36.0%. The mean CPQ11-14 score was 10.8 (SD 8.1) (Table 1). In the unadjusted analyses considering each predictor separately, SOC and bruxism variables were significantly associated with CPQ11-14 scores (Table 2).

**Table 1:** General sample characteristics (n = 406).

Variables		n* (%)
Sex		
	Boys	197 (48.5)
	Girls	209 (51.5)
Age		
	14	75 (18.9)
	15	83 (20.9)
	16	91 (22.9)
	17	148 (37.3)
Skin color		
	White	306 (77.1)
	Black, Brown, or Yellow	91 (22.9)
Household income in BMW		
	> 1	279 (77.7)
	≤ 1	85 (23.3)
Sense of coherence		
	High	171 (42.3)
	Low	233 (57.7)
Sleep bruxism		
	Without	361 (89.1)
	With	44 (10.9)
Awake bruxism		
	Without	279 (68.9)
	With	126 (31.1)
Dental caries		
	Absent	260 (64.0)
	Present	146 (36.0)
	Outcome	Mean (SD)
	CPQ11-14	10.8 (8.1)

SD: standard deviation; BMW: Brazilian minimum wage; CPQ11-14: child perception questionnaire; CI: confidence interval.

* Values less than 406 are due to missing data.

**Table 2:** Unadjusted analysis between bruxism variables, SOC, and overall CPQ11-14 scores.

Variables		CPQ11-14	
		Non-adjusted	p-value
		RR (95% CI)	
Sense of coherence			
	High	1 (reference)	
	Low	1.94 (1.81–2.07)	< 0.001
Sleep bruxism			
	Without	1 (reference)	
	With	1.15 (1.05–1.26)	< 0.001
Awake bruxism			
	Without	1 (reference)	
	With	1.16 (1.09–1.24)	< 0.001

RR: rate ratio; CI: confidence interval; SOC: sense of coherence.

Table 3 presents the unadjusted and adjusted analyses of the interaction between SB, AB, and SOC on overall CPQ11-14 scores. Adolescents with SB and low SOC had CPQ11-14 scores 1.71 times higher than those with SB and high SOC (RR 1.71; 95% CI: 1.38–2.11). Similarly, among adolescents with AB, those with low SOC showed 1.76 times higher CPQ11-14 scores compared to their high SOC counterparts (RR 1.76; 95% CI: 1.45–1.86), indicating a greater negative impact on OHRQoL.

**Table 3:** Non-adjusted and adjusted analysis of the interaction of sleep bruxism with SOC (model 1) and awake bruxism with SOC (model 2) on overall CPQ11-14 scores.

Variables		Outcome			
		Non-adjusted		Adjusted	
		RR (95%CI)	p-value	RR (95%CI)*	p-value
Model 1 - Sleep bruxism					
SB x SOC			< 0.001		
	With - High	1 (reference)	0.116	1 (reference)	
	With - Low	1.72 (1.39-2.13)	< 0.001	1.71 (1.38-2.11)	< 0.001
	Without - High	0.85 (0.69-1.04)		0.86 (0.70-1.06)	0.736
	Without - Low	1.66 (1.37-2.02)		1.63 (1.34-1.98)	<0.001
Model 2 - Awake bruxism					
AB x SOC					
	With - High	1 (reference)		1 (reference)	
	With - Low	1.82 (1.60-2.06)	< 0.001	1.76 (1.55-2.01)	< 0.001
	Without - High	0.87 (0.76-0.99)	0.040	0.86 (0.75-0.99)	0.041
	Without - Low	1.72 (1.52-1.93)	< 0.001	1.64 (1.45-1.86)	< 0.001

RR: rate ratio; CI: confidence interval; SOC: sense of coherence; SB: sleep bruxism; AB: awake bruxism;

* Adjusted by sex, age, skin color, household income, and dental caries.

Mean CPQ11-14 scores were approximately twice as high among individuals with low SOC compared to those with high SOC, both in the SB and AB groups, reinforcing the moderating role of SOC. Specifically, high SOC appeared to buffer the negative effects of bruxism on OHRQoL, whereas low SOC amplified them (p < 0.001). The difference in predicted CPQ11-14 scores between SOC levels was slightly more pronounced for AB (6.11) than for SB (5.83), although the contrast was modest (Table 4).

**Table 4:** Predictive marginal effects and contrast analysis of CPQ11-14 scores according to different levels of SOC among individuals with sleep bruxism and awake bruxism.

Variable		Margin (95%CI)	p-value
Model 1 - With sleep bruxism			
	High SOC	8.19 (6.62–9.75)	< 0.001
	Low SOC	14.02 (12.70–15.34)	< 0.001
	Contrast analysis*	5.83 (3.78–7.88)	< 0.001
Model 2 - With awake bruxism			
	High SOC	7.96 (1.06–8.86)	< 0.001
	Low SOC	14.07 (13.20–14.94)	< 0.001
	Contrast analysis*	6.11 (4.85–7.36)	< 0.001

SOC: sense of coherence; CI: confidence interval.

* dy/dx for factor levels is the discrete change from the base level.

## Discussion

This study evaluated the moderating role of SOC in the association between sleep and awake bruxism and OHRQoL among adolescents. The findings support the hypothesis that SOC moderates the effect of bruxism on OHRQoL. Adolescents with bruxism presented significantly poorer OHRQoL and those with low SOC exhibited CPQ11-14 scores nearly twice as high, indicating a stronger detrimental effect. Although previous studies have investigated the association between bruxism and OHRQoL,^7–9,12–24^ the potential moderating role of SOC—a key psychosocial resource—has not yet been explored. These results suggest that SOC may act as a resilience factor, particularly important in buffering the psychosocial impacts of bruxism among adolescents.

Overall, the results indicate that adolescents with self-reported bruxism behavior (both SB and AB) experience a greater negative impact on their OHRQoL, particularly when they have low levels of SOC. This finding is consistent with the understanding that bruxism behavior—whether occurring during sleep or wakefulness—can compromise various aspects of daily functioning and well-being. In the case of SB, the association with emotional factors such as stress, anxiety, and certain personality traits has been widely reported.^7,28,29^ This condition is often understood as an unconscious mechanism for releasing accumulated stress, which may disrupt sleep quality and result in daytime fatigue, affecting mood, concentration, and the ability to engage in daily activities. These consequences may amplify individuals’ perception of impaired oral health. Similarly, AB can result in discomfort, pain in the masticatory muscles, and functional limitations.^8,14^. These physical symptoms, combined with esthetic concerns such as dental wear or changes in facial musculature, may be particularly distressing for adolescents, a group especially sensitive to appearance and peer perception.^30^.

Importantly, our findings suggest that SOC plays a key moderating role in this context. Adolescents with self-reported SB and AB and a lower SOC presented poorer OHRQoL. In contrast, those with high SOC reported better OHRQoL despite the presence of bruxism, indicating that this psychosocial resource may buffer the subjective burden of bruxism-related symptoms. This reinforces the importance of considering individual coping resources in understanding and managing the impact of bruxism in adolescence. SOC is a psychological construct that reflects an individual's ability to perceive life as comprehensible, manageable, and meaningful, being associated with better coping strategies, resilience, and overall well-being.^15,16,18^ Different pathways can explain this finding, both at biochemical levels and in relation to individual behaviors.

One possible explanation for the protective effect of SOC on the relationship between bruxism and OHRQoL lies in its well-documented role in stress regulation.^30^ Both SB and AB have been associated with emotional and psychological factors—particularly stress, anxiety, and emotional dysregulation.^5,31^ SB is considered a motor manifestation of autonomic arousal during sleep, while AB often reflects a more conscious or semi-conscious response to daily stressors and psychological tension. In this context, adolescents with a high SOC may exhibit more adaptive coping mechanisms to deal with emotional challenges,^18,27^ which in turn reduces the psychological burden that intensifies bruxism-related consequences.

A strong SOC has been linked to lower cortisol reactivity and better autonomic nervous system balance,^32^ potentially decreasing the frequency or severity of bruxism episodes and their associated outcomes—such as orofacial pain, muscle fatigue, or poor sleep quality. From a neurobiological perspective, high SOC has been associated with increased activity in the prefrontal cortex, which is crucial for regulating emotional responses and managing stress.^33,34^ This regulatory control may attenuate the hyperactivation of the hypothalamic-pituitary-adrenal (HPA) axis^32,34^ thereby reducing the physiological arousal that contributes to both types of bruxism behavior.

Our findings also showed that the moderating effect of SOC was slightly more pronounced for AB compared to SB. This difference may stem from the fact that AB is more directly influenced by conscious emotional and behavioral responses to stress.^4,5,31^ As such, adolescents with low SOC—who may lack the cognitive and emotional resources to manage daily stress effectively—could be more vulnerable to and perceive persistent AB behaviors. These behaviors, unlike the automatic nature of SB, are more strongly modulated by psychological states and habits during waking hours, making them more sensitive to individual coping capacities.^4^

Furthermore, SOC is associated with engagement in health-promoting behaviors. Adolescents with a higher SOC are more likely to practice self-care, follow dental guidance, and seek professional help when facing bruxism-related complications.^35,36^ This proactive attitude can mitigate the development of secondary oral health issues, such as dental wear or temporomandibular disorders, which are known to negatively influence OHRQoL.^36,37^ Moreover, individuals with a stronger SOC may demonstrate greater psychological resilience and adaptability^15^, allowing them to interpret and manage their symptoms with less distress, which contributes to a more positive perception of their oral health—even in the presence of bruxism.

Some limitations of this study should be acknowledged. The cross-sectional design restricts the ability to infer causal relationships between bruxism, SOC, and OHRQoL. Although associations were observed, it is not possible to determine whether a low SOC precedes or results from the experience of self-reported SB, self-reported AB, and their impacts. In the same way, while we analyzed the influence of bruxism on OHRQoL, it is also plausible that adolescents with a negatively impacted quality of life might develop bruxism-like behaviors as a response to stress or emotional burden. Therefore, the possibility of reverse causality cannot be ruled out. Additionally, both SB and AB were assessed through self-report, which may introduce information bias when compared to clinical examination or instrumental measures^4^. Nonetheless, self-reported bruxism has been widely used in population-based studies^13,38^ and remains a practical and validated approach in epidemiological research^4^, especially considering the complexity, cost, and limited feasibility of instrumental measures in large samples. Finally, we did not collect information on menarche, and although most participants (14–18 years old) were likely to have already experienced it, this omission limits our ability to consider possible residual effects of pubertal hormonal changes.

Despite these limitations, our study has remarkable strengths. It is the first to examine the moderating role of SOC in the relationship between both SB and AB and OHRQoL in adolescents—a critical age group undergoing intense developmental, emotional, and social changes that may have long-term health consequences. By investigating psychosocial protective factors such as SOC, this study advances the understanding of how individual coping resources can buffer the adverse effects of stress-related behaviors like bruxism. Thus, SOC emerges as a relevant and modifiable factor that may help reduce the negative consequences of bruxism behaviors. Its influence on stress regulation, behavioral responses, and neurobiological mechanisms provides a plausible explanation for its protective role. Future longitudinal studies should explore these pathways further and evaluate the effectiveness of interventions aimed at strengthening SOC as a strategy to improve OHRQoL in adolescents affected by both SB and AB.

From a clinical perspective, our findings underscore the importance of considering adolescence as a vulnerable stage in which bruxism behaviors may be intertwined with psychosocial factors and quality of life. Given that the transition between pediatric and adult dental care is often not clearly defined, these results highlight the need for an interdisciplinary approach that integrates dental, psychological, and behavioral care. Such integration may contribute to more comprehensive strategies for prevention, early identification, and management of bruxism and its potential impacts during this critical period of development.

## Conclusion

Our findings showed that that both SB and AB are associated with worse OHRQoL in adolescents, and that a lower SOC significantly increases this negative impact—particularly in cases of AB. These findings highlight SOC as a relevant psychosocial resource, potentially modulating how adolescents perceive and cope with bruxism-related symptoms.

## Data Availability

The datasets generated during and/or analyzed during the current study are available from the corresponding author on reasonable request.
